# Ethyl acetate extracts of alfalfa (*Medicago sativa *L.) sprouts inhibit lipopolysaccharide-induced inflammation *in vitro *and *in vivo*

**DOI:** 10.1186/1423-0127-16-64

**Published:** 2009-07-14

**Authors:** Yong-Han Hong, Wen-Wan Chao, Miaw-Ling Chen, Bi-Fong Lin

**Affiliations:** 1 Department of Biochemical Science and Technology, Institute of Microbiology and Biochemistry, College of Life Science, National Taiwan University, Taipei, Taiwan, Republic of China; 2 Department of Medical Nutrition, I-Shou University, Kaohsiung County, Taiwan, Republic of China; 3 Department of Health and Nutrition, Chang Jung Christian University, Tainan County, Taiwan, Republic of China

## Abstract

This study aimed to investigate if food components that exert anti-inflammatory effects may be used for inflammatory disorders by examining alfalfa sprout ethyl acetate extract (ASEA). The cytokine profile and life span of BALB/c mice with acute inflammation after intra-peritoneal (ip) injection of 15 mg/kg BW lipopolysaccharide (LPS) were determined. The results showed that the life span of LPS-induced inflammatory mice were negatively correlated with serum levels of TNF-α, IL-6, and IL-1β at 9 hr after LPS-injection, which indicated that suppressing these cytokines in the late phase of inflammation may be beneficial for survival. The *in vitro *experiment then showed that ASEA significantly reduced IL-6 and IL-1β production and the NF-κB trans-activation activity of mitogen-stimulated RAW264.7 cells. To further evaluate the anti-inflammatory effects of ASEA *in vivo*, BALB/c mice were tube-fed with 25 mg ASEA/kg BW/day in 50 μl sunflower oil, while the control and PDTC (pyrrolidine dithiocarbamate, an anti-inflammatory agent) groups were tube-fed with 50 μl sunflower oil/day only. After one week of tube-feeding, the PDTC group was injected with 50 mg/kg BW PDTC and one hour later, all of the mice were injected with 15 mg/kg BW LPS. The results showed that the ASEA and PDTC groups had significantly lower serum TNF-α, IL-6, and IL-1β levels at 9 hr after LPS challenge, and significantly higher survival rates than the control group. This study suggests that ASEA supplementation can suppress the production of pro-inflammatory cytokines and alleviate acute inflammatory hazards.

## Background

There is growing evidence that systemic inflammation is associated with increased risk of chronic diseases such as cardio-vascular disease, cancer, and insulin resistance [1-3]. The mechanisms may involve macrophage and T lymphocyte activation, as well as release of pro-inflammatory cytokines that amplify the inflammatory activity [4,5]. Inflammation can be mediated by pro-inflammatory mediators, including tumor necrosis factor (TNF)-α, interleukin (IL)-1, IL-6, interferon (IFN)-γ, IL-12, IL-18, nitric oxide, and cell adhesion molecules [6]. These mediators help the innate immune response but their overproduction results in acute phase endotoxemia that causing tissue injury, organ failure, shock, and even death [7,8].

Among these mediators, pro-inflammatory cytokines such as TNF-α, IL-6, and IL-1β, are activated through NF-κB but they also activate NF-κB, thus amplifying the cytokine cascade and expanding the inflammatory status [9,10]. Because they play a driving role in the inflammatory process, effectively modulating their aberrant production can be beneficial in reducing inflammatory diseases. Therefore, the application of dietary components aside from anti-inflammatory drugs has recently become a focus of interest [11-13]. Dietary strategies have been recently proposed, including an anti-inflammatory diet for the prevention of chronic diseases associated with inflammation [14].

Alfalfa (*Medicago sativa *L.) sprouts are often consumed as vegetable salad. Its leaves or seeds are also sold as bulk powdered herb, capsules, and tablets for nutritional supplement in health food stores [15]. The extracts from alfalfa sprouts, leaves, and roots have been indicated to be helpful in lowering cholesterol levels in animal and human studies [16-18]. In addition, traditional medicinal use of alfalfa sprouts or leaves includes treatment of arthritis, kidney problems, and boils [19,20]. However, these treatments still need to be scientifically examined. Our previous study showed that the ethyl acetate extract of alfalfa sprouts ameliorates the autoimmune-prone disease of lupus mice, probably by attenuating cytokine and inflammatory responses [21]. Therefore, this study aimed to investigate the anti-inflammatory effect of the ethyl acetate extract of alfalfa sprouts with *in vitro *cell culture and through *in vivo *LPS-induced inflammatory mice.

## Materials and methods

### Reagents and materials

The reagents lipopolysacharide (LPS, E. coli serotype O55:B5), pyrrolidine dithiocarbamate (PDTC), and concanavalin (Con) A (Sigma Chemical Company; St. Louis, MO, USA) were all analytical grade and dissolved in phosphate buffer saline as a stock. Fresh alfalfa sprouts (*Medicago sativa L*.) (Goboul-Grange LTD; Taipei, Taiwan) were freeze-dried and finely grounded into the powder. The proximate composition of this powder was 33.6% carbohydrate, 49.6% protein, 2.6% lipid, 10.5% water, and 3.7% ash. Alfalfa sprout powders were extracted with ethyl acetate (EA 1:40, w/v, g/ml) by stirring at room temperature for 2 days. The sampled solvent solutions were collected and filtered through filter paper (Whatman No. 2; Whatman Paper Ltd, Maidstone). The alfalfa sprouts ethyl acetate extract (ASEA) was obtained by removing the solvent in a rotary evaporator, with a yield of 43.1 mg/g, and stored at -20°C.

### Cell culture experiment

The RAW264.7 cells (Bioresource Collection and Research Center; Hsinchu, Taiwan) were cultured in DMEM containing 10% fetal bovine serum (FBS), 2 mM glutamine, 1% non-essential amino acid, and 1 mM sodium pyruvate, and maintained in a humidified incubator at 37°C in 5% CO_2_. In this experiment, the RAW264.7 cells were seeded on 6-cm dishes at cell density of 2 × 10^6 ^cells/ml for NF-κB binding activity and mRNA expression, or 96-well plates at cell density of 2 × 10^4 ^cells/200 μl/well. The cells were then pre-treated with various concentrations of ASEA or PDTC (100 μM) for 1 hr before adding 100 ng/ml LPS for stimulated incubation. This PDTC concentration was shown to suppress cytokine production in RAW264.7 cells according to a previous report [22]. ASEA was dissolved in absolute ethanol for cell treatment (final ethanol concentration ≤ 0.1% in medium) and had no direct toxic effect on either RAW264.7 cells or primary macrophages.

For NF-κB binding activity and cytokine mRNA expression, the cells were lysed to obtain nuclear extracts and total RNA, respectively, after 24-hr incubation. For cytokine production, cell supernatants were collected for cytokine assay after 48-hr incubation, and cells at the bottom were assessed for viability.

For primary macrophage cell culture, peritoneal exudate cells isolated from BALB/c mice were seeded on 96-well plates at cell density of 2 × 10^5 ^cells/200 μl/well. Non-adherent cells were removed after 3 hr-incubation while adherent cells were pre-treated with various concentrations of ASEA (2, 10, and 50 μg/ml) 1 hr before addition of 10 μg/ml LPS for stimulation. Cell supernatants were collected for cytokine assay after 48-hr incubation. Three independent experiments were performed for all of the *in vitro *cell cultures.

### RNA isolation, reverse transcription and real-time PCR

The experiment was conducted according to a previous study [23]. RNA was isolated from RAW264.7 cells (> 5 × 10^6 ^cells) using the Trizol method (Invitrogen, Carlsbad, CA, USA) and reverse transcribed using 4 μg of total RNA, 10 mM dNTP (Takara, Shiga, Japan), 0.25 μg oligo dT (Promega, Madison, WI), 5 × M-MLV reaction buffer, and 200 U of M-MLV reverse transcriptase (Promega) in a final volume of 15 μl. The reaction was conducted for 1 hr at 42°C, followed by 5 min at 90°C, and slowly cooled to 4°C for 10 min.

The products were subjected to real-time PCR with primer sets of specific genes and SYBR Green PCR Master Mix (Bio-Rad Laboratories Inc., Hercules, CA, USA). The primer set used for mouse IL-6 was 5'-ACAGAAGGAGTGGCTAAGGAC-3' and 5'-GCTTAGGCATAACGCACTAGG-3'. The primer sets used as internal controls were 5'-CTATTGGCAACGAGCGGTTCC-3' and 5'-GCACTGTGTTGGCATAGAGGTC-3' for β-actin and 5'-TGTGTCCGTCGTGGATCTGAC-3' and 5'-GATGCCTGCTTCACCACCTTC-3' for GAPDH. The results were determined by a comparative *C*t value and analyzed with the iCycler iQ real-time PCR detection system (Bio-Rad Laboratories Inc.). The *C*t value of the control was used as a reference. All PCR-reactions were run in duplicate.

### NF-κB-dependent reporter assays

Transcriptional activation in the NF-κB binding site was assayed using a reporter plasmid, 3x-κB-tk-luciferase, containing three copies of the κB binding site in the upstream thymidine kinase (tk) promoter and a luciferase reporter gene in the pRL vector [24] (Dr. M. Kashiwada, NIH, Japan). Briefly, RAW264.7 cells were seeded on a 24-well plate at 1 × 10^5 ^cells/ml/well. Adherent cells were cultured for 18 hr and then transfected with 0.2 μg 3x-κB-tk-luciferase and 0.1 μg pRL-tk-luciferase (2:1) in 100 μl serum-free medium OPTI-MEM (Gibco BRL, Gaithersburg, MD, USA) containing 1 μl Lipofectamine™ 2000 (Invitrogen) per well. After 5 hr, the medium was changed to serum-free OPTI-MEM containing 100 ng/ml LPS and 1000 units/ml IFN-γ (Sigma) in the absence or presence of the different concentrations of ASEA. After 8 hr-incubation, the supernatants were removed and the luciferase reporter gene was analyzed.

Luciferase reporter assays were carried out according to the manufacturer's instructions [25]. Light emission was measured in a luminescence micro-plate counter (Wallac Victor-2 Perkin Elmer, Norwalk, MT, USA). Luciferase activity, expressed in arbitrary light units, was corrected for protein concentration in the sample, followed by normalization of transfection efficiency by β-galactosidase activity. The experiments were repeated thrice and showed the same tendency.

### Detection of NF-κB DNA-binding activity

Nuclear protein extracts and NF-κB DNA-binding activity in LPS-stimulated RAW264.7 cells were conducted by commercial kit (Active Motif, Carlsbad, CA, USA). Cells were washed twice with PBS/phosphatase inhibitor buffer and cell pellets were collected. Cells were re-suspended in hypotonic buffer on ice, and then reacted with Nonidet P-40. Nuclei were pelleted by centrifugation at 14000 × *g *for 30 sec. The pellet containing nuclei was re-suspended in extraction buffer (with protease inhibitor) and gently rocked on a shaking platform. By centrifugation at 14000 × *g *for 10 min, supernatants containing nuclear proteins were collected.

Protein concentrations of the nuclear lysates were determined using bicinchoninic acid assay. (Pierce Chemical Co., Rockfold, IL, USA). The DNA-binding activity of the p65 subunit of NF-κB in the nuclear protein extract was assessed using NF-κB p65 TransAM assay according to the manufacturer's instructions (Active Motif). Briefly, oligonucleotides containing the NF-κB consensus sequence were immobilized on a 96-well plate, which was incubated with the indicated nuclear protein extracts (2.5 μg/sample) for 1 hr at room temperature and shaken at 100 rpm. The plate was then washed and incubated with antibodies against p65 for 1 hr. After extensive washes, the plate was subsequently incubated with peroxidase-conjugated secondary antibodies for 1 hr. The peroxidase activity was visualized by tetramethylbenzidine reaction and the absorbance at 450 nm was determined using ELISA reader (EL311, Bio-TEK Inc., Winooski, VT, USA).

### Animal experiment and LPS-challenged acute inflammation

Five- to 6-week-old-female BALB/c mice from the Animal Center of the College of Medicine at National Taiwan University (Taipei, Taiwan) were maintained in an air-conditioned room at 23 ± 2°C on a regulated 12-hr light-dark cycle and housed in stainless steel cages with glass water bottles, and fed AIN-76 diet [26]. At age of 10 wks, the mice were started on experimental treatment or diet supplementation. Animal care and handling conformed to the National Institutes of Health's *Guide for the Care and Use of Laboratory Animals *[27].

To obtain the serum cytokine profile during acute inflammation, thirty 10-week-old BALB/c mice were injected intra-peritoneally (ip) with 15 mg/kg body weight (BW) LPS. Sera were obtained from 3–4 mice by retro-orbital bleeding at each time point of 0, 1.5, 2, 4, 6, 9, 12, and 24 hr after LPS challenge without repeat bleeding from the same mouse. To investigate the correlation between serum pro-inflammatory cytokine levels and life span, sera from sixty-six 10-week-old BALB/c mice were collected at 2 and 9 hr after 15 mg/kg BW LPS challenge, and the life spans of these mice were recorded.

### ASEA supplement prior to LPS-induced acute inflammation

Forty 10-week-old BALB/c mice were divided into three groups: the control (n = 15) and PDTC (n = 9, as a positive control) groups were tube-fed with 50 μl sunflower oil (SO)/day, while the ASEA group (n = 16) were tube-fed with 25 mg/kg BW ASEA in 50 μl SO/day. This oral dose of ASEA was derived from the effective dose (50 μg/ml) in primary peritoneal macrophage according to a previous report [28]. The PDTC group had fewer mice because this positive control group has been repeated and showed similar results in different experiments in our laboratory. The mice had free access to AIN-76 diet and water and were additionally tube-fed with either SO or ASEA daily. After one week of tube feeding, all of the mice were injected ip with 15 mg/kg BW LPS to induce acute inflammation. Mice in the PDTC group were injected ip with 50 mg/kg BW PDTC, a dose with anti-inflammatory effects as reported, 1 hr before LPS challenge [29,30]. Sera were collected at 2 and 9 hr after LPS challenge for cytokine assay. The life spans of mice that continued on AIN-76 diet *ad lib *were recorded.

### Assay of cytokine production

The production of cytokines TNF-α, IL-6, and IL-1β in the cell supernatants and mice serum was assayed by commercial ELISA kit. Briefly, anti-IL-6 (BD Pharmingen, San Diego, CA, USA), TNF-α, or IL-1β (R and D, Minneapolis, MN, USA) antibodies were coated to 96-well plates. After incubation, the wells in the plate were washed and blocked with blocking solution (PBS buffer containing 1% bovine serum albumin; Sigma), and then washed and properly diluted supernatant or serum was added. After incubation, the wells were washed and biotin-conjugated anti-IL-6, TNF-α, or IL-1β antibodies were added for 2 hr. The wells were then washed and horseradish peroxidase-conjugated streptavidin (Pierce Chemical Co.) was added for 30 min, washed, and incubated with ABTS (2, 20-azino-bis (3-ethylbenzthazoline-6-sulfonic acid), Sigma) or TMB (tetramethylbenzidine; Clinical Science Products, Mansfield, MA, USA). Absorbance at 405 nm or 620 nm was measured using ELISA reader (EL311, Bio-TEK Inc.). The data were calculated according to the cytokine standard curve.

### Statistical analysis

Data were expressed as mean ± SD or mean ± SEM. Significant difference compared to the control group was analyzed by the Student's *t*-test using the SAS software program (SAS/STAT version 8.0; SAS Institute, Cary, NC, USA). The relationship between serum pro-inflammatory cytokine level and survival was analyzed by simple correlation of the SAS program. Statistical comparison between different survival curves was analyzed by COX's proportion hazards regression test (STATA version 6.0; Stata Corp., College Station, TX, USA). A *P *value less than 0.05 was considered statistically significant.

## Results

### Serum pro-inflammatory cytokine release in LPS-challenged mice

**Figure 1:**
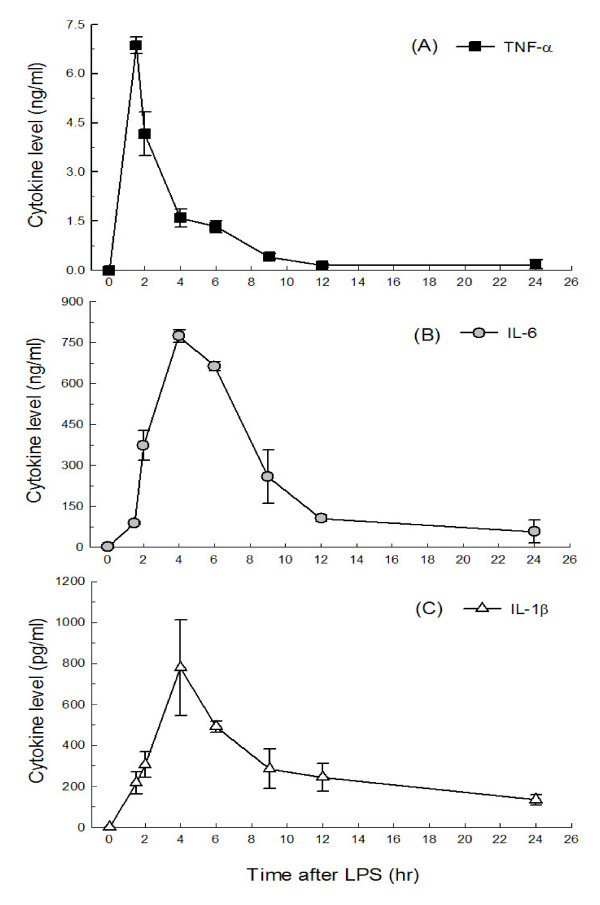
**Time-course of the production of pro-inflammatory cytokines, TNF-α, IL-6, and IL-1β, in the serum in BALB/c mice with LPS challenge (intra-peritoneal injection 15 mg/kg BW per mouse)**. Each time point value was an average of data from 3–4 mice and shown as mean ± SEM.

To investigate changes of cytokines in acute inflammation, serum levels of pro-inflammatory cytokines were determined at different time points after 15 mg/kg BW LPS challenge in BALB/c mice. Serum TNF-α level peaked at 1.5 hr (6.86 ± 0.25 ng/ml) and then quickly declined to the base level over 12 hr (0.16 ± 0.05 ng/ml) (Figure 1A). Serum IL-6 and IL-1β reached the highest levels at 4 hr (774 ± 22 ng/ml and 783 ± 234 pg/ml), dropped at 9 hr, and remained detectable at 24 hr (57.1 ± 41.7 ng/ml and 135 ± 25 pg/ml) (Figures 1B and 1C).

### Negative correlation between life span and serum cytokine levels in acute inflammation

**Figure 2:**
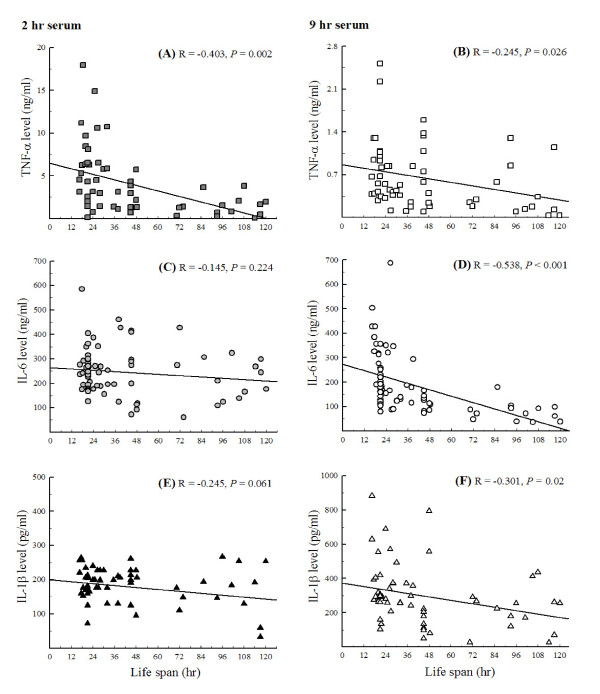
**The correlation between serum cytokine levels (at two time points, 2 hr and 9 hr, after LPS injection) and life span in BALB/c mice with LPS challenge**. This correlation was analyzed by the simple correlation in SAS 8.0 software (n = 64, *p *< 0.05).

In order to investigate the association of serum cytokine level in the early and late phases of acute inflammation with life span of LPS-induced inflammatory mice, sera at 2 and 9 hr after LPS-challenge were obtained for TNF-α, IL-6, and IL-1β measurement. The correlation between life span and serum cytokine level was analyzed (Figure 2). At 2 hr after LPS-challenge, only serum TNF-α level negatively correlated with life span (r = -0.403, *p *= 0.002), while at 9 hr, serum levels of TNF-α, IL-6, and IL-1β all negatively correlated with life span (r = -0.295, *p *= 0.026 for TNF-α; r = -0.538, *p *< 0.001 for IL-6; r = -0.301, *p *= 0.022 for IL-1β).

### ASEA inhibited pro-inflammatory cytokine production from LPS-stimulated RAW264.7 cells and peritoneal macrophages

In the preliminary test for anti-inflammatory effect of alfalfa sprouts, extract in ethyl acetate rather than methanol or hexane showed suppressive effects on TNF-α, IL-6, and IL-1β production by LPS-stimulated primary macrophage cells (data not shown). This was the rationale for selecting alfalfa sprout extract in ethyl acetate (ASEA) for further study. The pre-treatment of RAW264.7 cells and primary peritoneal macrophages with 2, 10, 50 μg/ml ASEA or 100 μM PDTC for 1 hr had no significant effect on cell viability (data not shown).

**Figure 3:**
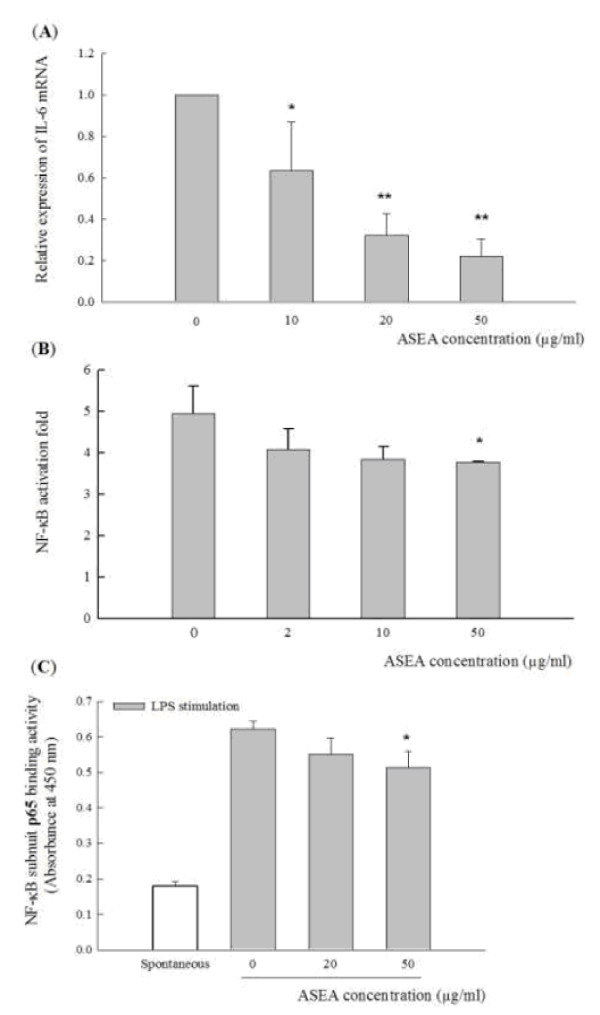
**Effects of ASEA on IL-6 mRNA expression and NF-κB activation in LPS-stimulated RAW264.7 cells**. **(A) **Cells pre-treated with 0 (control), 10, 20 or 50 μg/ml ASEA for 1 hr were stimulated with LPS for IL-6 mRNA expression by real-time PCR. **(B) **Cells transfected with the 3x-κB-tk-luciferase and pRL-tk-luciferse plasmids were stimulated with LPS and IFN-γ in the absence (0) or the presence of 2, 10, and 50 μg/ml ASEA for the reporter assay. **(C) **Nuclear extracts from unstimulated (spontaneous) or LPS-stimulated cells pre-treated with 0, 20, or 50 μg/ml ASEA were for DNA-binding activities of the NF-κB subunit, p65. Bar values are mean ± SD in three independent experiments in these assays. A significant difference from the control was indicated as *p < 0.05 or **p < 0.01 by Student's *t*-test.

As shown in Table 1, the anti-inflammatory reagent PDTC, as a positive control, suppressed TNF-α and IL-6 production at a concentration of 100 μM (90% and 55% inhibition, respectively). ASEA at a concentration of 50 μg/ml significantly inhibited IL-6 and IL-1β production in both LPS-stimulated RAW264.7 cells (51% and 63% inhibition, respectively) and peritoneal macrophages (55% and 38% inhibition, respectively). Further investigation of mRNA expression also showed lower IL-6 mRNA in LPS-stimulated RAW264.7 cells pre-treated with 50 μg/ml ASEA (78% inhibition) (Figure 3A).

**Table 1:** Effects of ASEA on pro-inflammatory cytokines production from LPS-stimulated RAW264.7 cells and peritoneal macrophages.

Treatment	RAW 264.7, ng/ml^a^			Macrophages, ng/ml^b^		
	
	IL-1β	IL-6	TNF-α	IL-1β	IL-6	TNF-α
Control (0)	0.16 ± 0.02	36.3 ± 1.61	12.3 ± 2.63	1.22 ± 0.22	36.3 ± 5.75	1.67 ± 0.21
ASEA 2 μg/ml	0.19 ± 0.01	32.1 ± 0.62	10.9 ± 4.01	1.13 ± 0.24	41.6 ± 9.12	1.77 ± 0.25
ASEA 10 μg/ml	0.17 ± 0.02	20.8 ± 2.88*	14.6 ± 2.71	1.06 ± 0.32	34.6 ± 6.68	1.44 ± 0.29
ASEA 50 μg/ml	0.06 ± 0.01*	17.4 ± 5.81**	14.3 ± 1.72	0.76 ± 0.24*	16.4 ± 5.55*	1.41 ± 0.15
PDTC100 μM	0.14 ± 0.03	15.9 ± 3.24**	1.58 ± 0.39**		N.D.^c^	

Values are expressed as means ± SD of three independent experiments with triplicates each and statistically analyzed by using Student's *t*-test. A significant difference from the control was indicated *p < 0.05.

^a^RAW264.7 cells were stimulated 100 ng/ml LPS for 48-hr incubation.

^b^Murine peritoneal macrophages were stimulated with 10 μg/ml LPS for 48-hr incubation.

^c^N.D. means no detection in cytokine profile of peritoneal macrophages.

To test whether ASEA pre-treatment may involve NF-κB activation, a reporter gene assay with 3x-κB-tk-luciferase after transfection into RAW264.7 cells was performed (Figure 3B). Pre-treatment of RAW264.7 cells with ASEA suppressed NF-κB activation, significantly lower at concentration of 50 μg/ml (23% inhibition). The p65 subunit of NF-κB, also known as RelA, contained an important trans-activating domain for NF-κB-mediated gene activation [25]. Its significantly lower binding activity was also observed (Figure 3C).

### ASEA reduced serum levels of pro-inflammatory cytokines in LPS-challenged mice

For the *in vivo *study, mice were tube-fed 25 mg/kg BW/day ASEA for one-week and challenged with LPS to induce acute inflammation. Sera were collected at 2 and 9 hr after LPS-challenge for pro-inflammatory cytokine assay (Table 2). The PDTC positive control group had significantly lower serum levels of cytokines, except for IL-6 level at 2 hr after LPS challenge, compared to the control group. The ASEA group also had lower serum levels of TNF-α, IL-6, and IL-1β, especially significant at 9 hr after LPS injection compared to the control.

**Table 2:** The effect of ASEA supplement on the production of pro-inflammatory cytokines in serum of LPS-challenged BALB/c mice.

Cytokine	Time after LPS	Control	ASEA	PDTC
		
		n = 15	n = 16	n = 9
		
		Serum cytokine levels, ng/ml		
TNF-α	2 hr	7.51 ± 1.37	5.63 ± 1.01	0.87 ± 0.32 **
	9 hr	0.63 ± 0.09	0.31 ± 0.06 *	0.16 ± 0.03 *
IL-6	2 hr	286 ± 28	235 ± 15	299 ± 19
	9 hr	307 ± 43	145 ± 26 **	49 ± 43 **
IL-1β	2 hr	0.21 ± 0.01	0.19 ± 0.01	0.18 ± 0.01 *
	9 hr	0.38 ± 0.05	0.23 ± 0.03 *	0.12 ± 0.03 **

Sera at 2 hr and 9 hr after LPS injection were collected for cytokines assay. The cytokine production in serum was assayed by ELISA. Values are means ± SEM. *p < 0.05, **p < 0.01, significantly different from the control group analyzed by Student's *t*-test.

### ASEA supplement benefited survival of LPS-challenged mice

**Figure 4:**
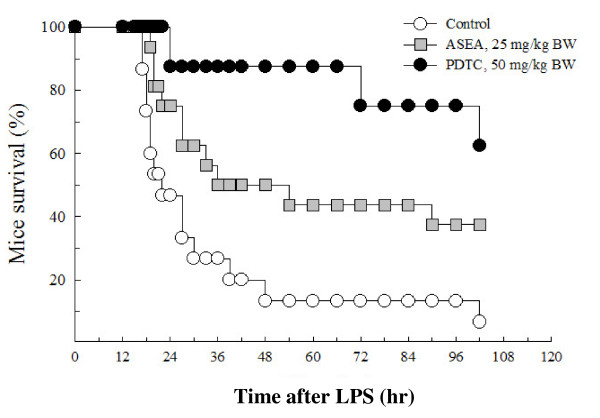
**Effects of ASEA supplementation on the survival of mice challenged with 15 mg/kg BW LPS**. The ASEA and PDTC positive control groups had increased survival using the analysis of COX's proportion hazards regression test (*p *= 0.03 and *p *= 0.01, respectively).

To investigate whether suppression of pro-inflammatory cytokine production may benefit survival of LPS-challenged mice, the life spans were recorded (Figure 4). Only half of the mice survived at 24 hr after LPS challenge in the control group, but approximately three-fourths (12 of 16 mice) in the ASEA group survived. At 48 hr, 50% (8/16) in the ASEA group survived as compared to 13% (2/15) in the control group. The survival rate in the ASEA group was significantly higher than that of the control group according to the COX's proportion hazards regression test (hazard ratio = 0.37, *p *= 0.03). The PDTC group also had a higher survival rate (hazard ratio = 0.07, *p *= 0.01).

## Discussion

In normal conditions without any stimuli or treatment, serum TNF-α and IL-1β levels are barely detectable, while IL-6 level at 1~2 ng/ml is detectable in BALB/c mice [31]. After LPS challenge, serum TNF-α levels quickly rises, followed by IL-1β and IL-6 [32]. However, the induced elevation and release of these pro-inflammatory cytokines may vary according to the animal species and LPS dosage used. Most studies use low dose LPS (5 mg/kg BW) for cytokine profile and high doses (35~44 mg/kg BW) for survival studies [29,31,33]. The current study utilized a sub-lethal dose of 15 mg/kg BW according to a dose titration study showing mice with 100% survival at 14 hr and 50% survival at 24 hr after LPS challenge, which allows for examining the profile of cytokines not only at the early but also at the late stage of LPS-induced acute inflammation.

This study is the first to illustrate the cytokine profile of BALB/c mice with 15 mg/kg BW LPS challenge (Figure 1). Data showed that peak levels of serum TNF-α are similar, but the several hundred-fold increase of IL-6 and IL-1β were different from other studies [29,31,33]. Serum TNF-α peaked at 1.5 hr after LPS injection and at 1 or 1.5 hr in studies with similar levels (~7 ng/ml) despite varying mice species and LPS dosage [34]. This suggests that TNF-α is the pro-inflammatory cytokine that responds most rapidly, reaches certain peak levels in blood, and drops after exerting its function [29,32]. Serum IL-6 showed higher peak levels and peaked at 4 hr after LPS challenge in our study rather than 2~3 hr with various peak levels in other studies [33,35]. Serum IL-1β levels were higher and reached peak levels at 4 hr after LPS challenge in our study compared to those of others studies peaking at 2~6 hr with the same LPS dosage but different mice species [31,33,34]. Therefore, overall, the time point of 2 hr after LPS challenge was chosen for further investigation of the correlation between early cytokine production and life span.

In addition, there were detectable sera TNF-α, IL-6, and IL-1β at 9 hr after LPS challenge with a dose of 15 mg/kg BW, which was different from other studies in which these cytokines were almost undetectable when 5 mg/kg BW was used. This provides further insight into the anti-inflammatory effect in late stage LPS-induced acute inflammation. The findings here suggest that the LPS dose of 15 mg/kg BW is an optimal challenge dose for studying both cytokine profile and survival. The effects elicited by this dose can be modulated by food components.

Survival rate in murine endotoxic shock has been used as an indicator of anti-inflammatory effect. However, it remains unclear if the early or late evocation of serum TNF-α, IL-1β, and IL-6 is crucial for survival. In the current study, cytokine levels in serum obtained at 2 and 9 hrs after LPS challenge and life spans were followed using the same batch of mice to evaluate the association of serum cytokine levels and survival (Figure 2). The results show that TNF-α level at 2 hr negatively correlates with life span, suggesting that down-regulating serum TNF-α in the early stage of acute-inflammation may prolong the life of mice suffering from endotoxic shock by LPS injection. Suppression of TNF-α production by some plant extracts, such as *Dioscoreae Rhizoma*, *Prunella vulgaris*, and *Salvia miltiorrhiza bunge *is suggested to ameliorate several inflammatory disorders, including rheumatoid arthritis, endotoxemia, and anaphylaxis [35-37]. Potential mechanisms have been proposed to inhibit signal transductions, such as protein kinase C, tyrosine kinase, and activation of NF-κB [38,39].

Interestingly, IL-6, IL-1β, or TNF-α level at 9 hr is also negatively correlated with life span. This implies that inhibiting the subsequent production of pro-inflammatory cytokines (the later stage) still benefits animal survival. The correlation is consistent with the observation that one week pre-treatment of ASEA only significantly decreased serum levels of pro-inflammatory cytokines at 9 hr after LPS challenge but could still prolong life in inflammatory mice (Table 2). PDTC, known to inhibit NF-κB, has been demonstrated to protect mice from lethal endotoxic shock when ip injected 30 min or 1 hr prior to the induction [29,30]. PDTC, as the positive control group in the present study, significantly suppressed serum TNF-α and IL-1β not only at 9 hr but also at 2 hr after LPS-challenge, and thus had the least mortality among the three groups (Figure 4).

As for anti-inflammation food materials, recent studies show that components from food sources such as apples, *Ganoderma tsugae*, and walnuts may provide anti-inflammatory activity and offer nutritional benefits in inflammation [14,40,41]. Therefore, dietary components and nutritional supplement have also been proposed to function in a manner that prevents certain inflammatory diseases, suggesting that more investigations on beneficial food components and mechanisms of actions are warranted. These food materials, including ASEA in this study, were administered orally to decrease inflammatory responses. It has been documented that mucosal immune mediators such as transforming growth factor-α (TGF-α) and IL-10 can exert a down-regulatory effect of immune response [42,43]. More studies are needed to confirm that oral delivery of ASEA activates certain immune effector cells and induces the immune response to down-regulate the inflammatory responses.

Macrophage cell lines, such as murine RAW264.7 cell and human THP-1 cells, have been used as rapid *in vitro *screening methods to study the anti-inflammatory agent [44]. In the present study, RAW264.7 cells and murine peritoneal macrophages were used for *in vitro *screening. PDTC, as a positive control, significantly decreased IL-6 and TNF-α, but not IL-1β production from LPS-stimulated RAW264.7 cells (Table 1). This is consistent with other reports though different cells like the J774A.1 murine macrophage-like cell line and peritoneal macrophages were used [29].

Similarly, 50 μg/ml ASEA pre-treatment also significantly suppressed two of the pro-inflammatory cytokines, IL-6 and IL-1β, but not TNF-α, in LPS-stimulated RAW264.7 cells. No significant suppression of TNF-α by ASEA pre-treatment might be due to TNF-α secretion reached the maximum at a shorter duration of incubation, and thus IL-6 and IL-1β were the more sensitive cytokines for significant suppression in RAW264.7 cells under 48 hr-incubation in our study [44]. Nevertheless, the dose of 25 mg/kg BW ASEA supplement for *in vivo *study derived from 50 μg/ml tested *in vitro *according to a previous report [28] also showed suppression of serum IL-6, IL-1β and TNF-α, which indicate consistent results from *in vitro *and *in vivo *anti-inflammatory screening strategies.

In addition, ASEA not only inhibits IL-6 secretion in a dose-dependent manner but also reduces mRNA expression (Figure 3). Consistently, ASEA suppresses NF-κB activation, suggesting that the suppressive effect of ASEA on pro-inflammatory cytokines may involve the inhibition of NF-κB activation [45]. The trans-activation assay by the transient transfection of luciferase reporter plasmid to the cell line is a tool for massive screening [46,47]. A NF-κB-dependent luciferase construct containing three copies of an NF-κB binding site was used in this study to further confirm the anti-inflammatory effect of ASEA. NF-κB binding sites are involved in the regulation of many genes, such as cytokines and chemokines [45]. The reduction of luciferase assay and NF-κB DNA-binding activity by ASEA confirms the anti-inflammatory effect of ASEA, and suggests that both murine macrophage cell lines and NF-κB-dependent luciferase report assays are efficient methods for screening in future studies.

Which component of ASEA exerts this anti-inflammatory effect is now under investigation. Though coumestrol, the major phytoestrogen in alfalfa sprouts, was also tested in the experiment, the results showed that TNF-α, IL-6, and IL-1β secretions from LPS-stimulated RAW264.7 cells were not affected by 5–500 nM coumestrol pre-treatment (data not shown). This may exclude coumestrol as an effective component. By bioactivity-guided chromatographic fractionation, the major bioactive compounds are loliolide, 4-hydroxy-6-nonadecyl-tetrahydro-pyran-2-one, and flavonoids such as liquiritigenin and isoliquiritigenin, that exerting significant inhibition of IL-6 and IL-1β secretions from LPS-stimulated peritoneal macrophages of BALB/c mice. The isolation and characterization of anti-inflammatory effect of these compounds are undergoing in our laboratory.

In conclusion, this study demonstrated that alfalfa sprouts extract in ethyl acetate significantly suppresses serum production of pro-inflammatory cytokines and increases survival in LPS-challenged mice.

## Competing interests

The authors declare that they have no competing interests.

## Authors' contributions

In this study, YHH collected all data, ran statistical reports and wrote the paper. BFL devised and revised the paper. WWC and MLC helped the animal care and some of the assays. All authors read and approved the final manuscript.

## References

[B1] Libby P (2006). Inflammation and cardiovascular disease mechanisms. Am J Clin Nutr.

[B2] Schottenfeld D, Beebe-Dimmer J (2006). Chronic inflammation-a common and important factor in the pathogenesis of neoplasia. CA Cancer J Clin.

[B3] Shoelson SE, Herrero L, Naaz A (2007). Obesity, inflammation, and insulin resistance. Gastroenterology.

[B4] Tousoulis D, Charakida M, Stefanadis C (2005). Endothelial function and inflammation in coronary artery disease. Heart.

[B5] Lin WW, Karin M (2007). A cytokine-mediated link between innate immunity, inflammation, and cancer. J Clin Invest.

[B6] Fairweather D, Rose NR (2005). Inflammatory heart disease: a role for cytokines. Lupus.

[B7] Bone RC (1996). The sepsis syndrome: Definition and general approach to management. Clin Chest Med.

[B8] Astiz ME, Rackow EC (1998). Septic shock. Lancet.

[B9] Collins T, Read MA, Neish AS, Whitley MZ, Thanos D, Maniatis T (1995). Transcriptional regulation of endothelial cell adhesion molecules: NF-κB and cytokine inducible enhancers. FASEB J.

[B10] Baldwin AS (1996). The NF-κB and IκB proteins: New discoveries and insights. Annu Rev Immunol.

[B11] Chan MM, Huang HI, Fenton MR, Fong D (1998). *In vivo *inhibition of nitric oxide synthase gene expression by curcumin, a cancer preventive natural product with anti-inflammatory properties. Biochem Pharmacol.

[B12] Paradkar PN, Blum PS, Berhow MA, Baumann H, Kuo SM (2004). Dietary isoflavones suppress endotoxin-induced inflammatory reaction in liver and intestine. Cancer Lett.

[B13] Davis PA, Polagruto JA, Valacchi G, Phung A, Soucek K, Keen CL, Gershwin ME (2006). Effects of apple extracts on NF-κB activation of human umbilical vein endothelial cells. Exp Biol Med (Maywood).

[B14] O'keefe JH, Gheewala NM, O'keefe JO (2008). Dietary strategies for improving post-prandial glucose, lipids, inflammation, and cardiovascular health. J Am Coll Cardiol.

[B15] Brinker F, Stodart N (2001). Herb contraindications and drug interactions. Eclectic Medical Publications.

[B16] Story JA, LePage SL, Petro MS, West LG, Cassidy MM, Lightfoot FG, Vahouny GV (1984). Interactions of alfalfa plant and sprout saponins with cholesterol in vitro and in cholesterol-fed rats. Am J Clin Nutr.

[B17] Molgaard J, von Schenck H, Olsson AG (1987). Alfalfa seeds lower low density lipoprotein cholesterol and apolipoprotein B concentrations in patients with type II hyperlipoproteinemia. Atherosclerosis.

[B18] Colodny LR, Montgomery A, Houston M (2001). The role of esterin processed alfalfa saponins in reducing cholesterol. J Am Nutraceutical Assoc.

[B19] Foster S, Duke JA, Peterson RT (1990). Eastern and Central North America. A Field Guide to Medicinal Plants and Herbs.

[B20] Barnes J, Anderson LA, Phillipson JD, Barnes J, Anderson LA, Phillipson JD (2002). A guide for health-care professionals. Herbal Medicines.

[B21] Hong YH, Huang CJ, Wang SC, Lin BF (2009). The ethyl acetate extract of alfalfa sprouts ameliorates autoimmune-prone disease of MRL-*lpr/lpr *mice. Lupus.

[B22] Lemay S, Lebedeva TV, Singh AK (1999). Inhibition of cytokine gene expression by sodium salicylate in a macrophage cell line through an NF-kappaB-independent mechanism. Clin Diagn Lab Immunol.

[B23] Jobin C, Haskill S, Mayer L, Panja A, Sartor RB (1997). Evidence for altered regulation of IκBα degradation in human colonic epithelial cells. J Immunol.

[B24] Kashiwada M, Shirakata Y, Inoue JI, Nakano H, Okazaki K, Okumura K, Yamamoto T, Nagaoka H, Takemori T (1998). Tumor necrosis factor receptor-associated factor 6 (TRAF6) stimulates extracellular signal-regulated kinase (ERK) activity in CD40 signaling along a Ras-independent pathway. J Exp Med.

[B25] Van den Berghe W, Plaisance S, Boone E, De Bosscher K, Schmitz ML, Fiers W, Haegeman G (1998). P38 and extracellular signal-regulated kinase mitogen-activated protein kinase pathways are required for nuclear factor kappaB p65 transactivation mediated by tumor necrosis factor. J Biol Chem.

[B26] American Institute of Nutrition (1997). Report of the American Institute of Nutrition Ad Hoc Committee on Standards for Nutritional Studies. J Nutr.

[B27] National Research Council (1985). Guide for the Care and Use of Laboratory animals.

[B28] Hong YH, Lin BF (2001). Study of the anti-inflammatory effect of rice shoots juice. Taiwanese J Agric Chem Food Sci.

[B29] Meisner M, Schmidt J, Schywalsky M, Tschaikowsky K (2000). Influence of pyrrolidine dithiocarbamate on the inflammatory response in macrophages and mouse endotoxin shock. Int J Immunopharmacol.

[B30] Liu SF, Ye X, Malik AB (1999). Inhibition of NF-kappa B activation by pyrrolidine dithiocarbamate prevents in vivo expression of pro-inflammatory genes. Circulation.

[B31] Blanque R, Meakin C, Millet S, Gardner CR (1996). Hypothermia as an indicator of the acute effects of lipopolysaccharides: Comparison with serum levels of IL-1, IL-6 and TNF-α. Gen Pharmacol.

[B32] Kotanidou A, Xagorari A, Bagli E, Kitsanta P, Fotsis T, Papapetropoulos A, Roussos C (2002). Luteolin reduces lipopolysaccharide-induced lethal toxicity and expression of pro-inflammatory molecules in mice. Am J Respir Crit Care Med.

[B33] Jones KL, Mansell A, Patella S, Scott B, Hedger MP, de Kretser DM, Phillips DJ (2007). Activin A is a critical component of the inflammatory response, and its binding protein, follistatin, reduces mortality in endotoxemia. Proc Natl Acad Sci USA.

[B34] Blanque R, Meakin C, Millet S, Gardner CR (1999). Dual mechanisms of action of interferon-γ potentiating responses to LPS in mice IL-1, TNF-α, and IL-6 production in serum and hypothermia. Gen Pharmacol.

[B35] Kim MJ, Kim HN, Kang KS, Baek NI, Kim DK, Kim YS, Jeon BH, Kim SH (2004). Methanol extract of *Dioscoreae Rhizoma *inhibits pro-inflammatory cytokines and mediators in the synoviocytes of rheumatoid arthritis. Int Immunopharmacol.

[B36] Wan JM, Sit WH, Lee CL, Fu KH, Chan DK (2006). Protection of lethal toxicity of endotoxin by *Salvia miltiorrhiza *BUNGE is via reduction in tumor necrosis factor alpha release and liver injury. Int Immunopharmacol.

[B37] Kim SY, Kim SH, Shin HY, Lim JP, Chae BS, Park JS, Hong SG, Kim MS, Jo DG, Park WH, Shin TY (2007). Effects of *Prunella vulgaris *on mast cell-mediated allergic reaction and inflammatory cytokine production. Exp Biol Med (Maywood).

[B38] Huang YT, Hwang JJ, Lee PP, Ke FC, Huang CJ, Kandaswami C, Middleton E, Lee MT (1999). Effects of luteolin and quercetin, inhibitors of tyrosine kinase, on cell growth and metastasis-associated properties in A431 cells overexpressing epidermal growth factor receptor. Br J Pharmacol.

[B39] Chen YH, Lin SJ, Ku HH, Shiao MS, Lin FY, Chen JW, Chen YL (2001). Salvianolic acid B attenuates VCAM-1 and ICAM-1 expression in TNF-alpha-treated human aortic endothelial cells. J Cell Biochem.

[B40] Lin JY, Chen ML, Lin BF (2006). *Ganoderma tsugae *in vivo modulates Th1/Th2 and macrophage responses in an allergic murine model. Food Chem Toxicol.

[B41] Papoutsi Z, Kassi E, Chinou I, Halabalaki M, Skaltsounis LA, Moutsatsou P (2008). Walnut extract (*Juglans regia L*.) and its component ellagic acid exhibit anti-inflammatory activity in human aorta endothelial cells and osteoblastic activity in the cell line KS483. Br J Nutr.

[B42] Chavali SR, Zhong WW, Utsunomiya T, Forse RA (1997). Decreased production of interleukin-1-beta, prostaglandin-E2 and thromboxane-B2, and elevated levels of interleukin-6 and -10 are associated with increased survival during endotoxic shock in mice consuming diets enriched with sesame seed oil supplemented with Quil-A saponin. Int Arch Allergy Immunol.

[B43] Mucida D, Park Y, Kim G, Turovskaya O, Scott I, Kronenberg M, Cheroutre H (2007). Reciprocal TH17 and regulatory T cell differentiation mediated by retinoic acid. Science.

[B44] Singh U, Tabibian J, Venugopal SK, Devaraj S, Jialal I (2005). Development of an in vitro screening assay to test the antiinflammatory properties of dietary supplements and pharmacological agents. Clin Chem.

[B45] Li Q, Verma IM (2002). NF-κB regulation in the immune system. Nat Rev Immunol.

[B46] Chao CY, Huang CJ (2003). Bitter gourd (*Momordica charantia*) extract activates peroxisome proliferator-activated receptors and upregulates the expression of the acyl CoA oxidase gene in H4IIEC3 hepatoma cells. J Biomed Sci.

[B47] Cheng WY, Kuo YH, Huang CJ (2007). Isolation and identification of novel estrogenic compounds in yam tuber (*Dioscorea alata Cv*. Tainung No. 2). J Agric Food Chem.

